# The effect of liquid hot water pretreatment on the chemical–structural alteration and the reduced recalcitrance in poplar

**DOI:** 10.1186/s13068-017-0926-6

**Published:** 2017-11-30

**Authors:** Mi Li, Shilin Cao, Xianzhi Meng, Michael Studer, Charles E. Wyman, Arthur J. Ragauskas, Yunqiao Pu

**Affiliations:** 1 BioEnergy Science Center (BESC), Oak Ridge, USA; 2 Biosciences Division, ORNL, Oak Ridge, TN USA; 3 School of Chemistry and Biochemistry, Georgia Institute of Technology, Atlanta, GA USA; 4 College of Engineering - Center for Environmental Research and Technology (CE-CERT), Bourns College of Engineering, University of California, Riverside, CA USA; 5 Department of Chemical and Biomolecular Engineering, University of Tennessee, Knoxville, TN USA; 6 Department of Forestry, Wildlife, and Fisheries, Center for Renewable Carbon, University of Tennessee Institute of Agriculture, Knoxville, TN USA; 7 Present Address: College of Material Engineering, Fujian Agriculture and Forestry University, Fuzhou, People’s Republic of China; 8 Present Address: Laboratory for Bioenergy and Biochemicals, School of Agricultural, Forest and Food Sciences, Bern University of Applied Sciences, Bern, Switzerland

**Keywords:** Poplar, Liquid hot water pretreatment, Biomass recalcitrance, Biopolymers structures

## Abstract

**Background:**

Hydrothermal pretreatment using liquid hot water (LHW) is capable of substantially reducing the cell wall recalcitrance of lignocellulosic biomass. It enhances the saccharification of polysaccharides, particularly cellulose, into glucose with relatively low capital required. Due to the close association with biomass recalcitrance, the structural change of the components of lignocellulosic materials during the pretreatment is crucial to understand pretreatment chemistry and advance the bio-economy. Although the LHW pretreatment has been extensively applied and studied, the molecular structural alteration during pretreatment and its significance to reduced recalcitrance have not been well understood.

**Results:**

We investigated the effects of LHW pretreatment with different severity factors (log *R*_0_) on the structural changes of fast-grown poplar (*Populus trichocarpa*). With the severity factor ranging from 3.6 to 4.2, LHW pretreatment resulted in a substantial xylan solubilization by 50–77% (*w/w*, dry matter). The molecular weights of the remained hemicellulose in pretreated solids also have been significantly reduced by 63–75% corresponding to LHW severity factor from 3.6 to 4.2. In addition, LHW had a considerable impact on the cellulose structure. The cellulose crystallinity increased 6–9%, whereas its degree of polymerization decreased 35–65% after pretreatment. We found that the pretreatment severity had an empirical linear correlation with the xylan solubilization (*R* ^2^ = 0.98, *r* = + 0.99), hemicellulose molecular weight reduction (*R* ^2^ = 0.97, *r* = − 0.96 and *R* ^2^ = 0.93, *r* = − 0.98 for number-average and weight-average degree of polymerization, respectively), and cellulose crystallinity index increase (*R* ^2^ = 0.98, *r* = + 0.99). The LHW pretreatment also resulted in small changes in lignin structure such as decrease of β-*O*-4′ ether linkages and removal of cinnamyl alcohol end group and acetyl group, while the S/G ratio of lignin in LHW pretreated poplar residue remained no significant change compared with the untreated poplar.

**Conclusions:**

This study revealed that the solubilization of xylan, the reduction of hemicellulose molecular weights and cellulose degree of polymerization, and the cleavage of alkyl–aryl ether bonds in lignin resulted from LHW pretreatment are critical factors associated with reduced cell wall recalcitrance. The chemical–structural changes of the three major components, cellulose, lignin, and hemicellulose, during LHW pretreatment provide useful and fundamental information of factors governing feedstock recalcitrance during hydrothermal pretreatment.

**Electronic supplementary material:**

The online version of this article (doi:10.1186/s13068-017-0926-6) contains supplementary material, which is available to authorized users.

## Background

The utilization of lignocellulosic materials for the production of bioenergy and bio-based materials has been noticeably progressed with the advancement in process chemistry, genetics, biotechnology, and engineering [1, 2]. However, the native recalcitrant properties of plant remain as a challenge for the efficient utilization of biomass employing the biochemical conversion pathway [3]. Biomass recalcitrance associated with the structural heterogeneity and complexity of the plant cell wall has been attributed to several factors such as lignification, cellulose and hemicellulose structure, and lignin–carbohydrate complex (LCC) linkages [4, 5], making pretreatment an essential prerequisite to overcome biomass recalcitrance and to achieve the conversion efficiency to cellulosic ethanol.

Among the various pretreatment methods, liquid hot water (LHW) pretreatment has become one of the leading pretreatment technologies utilizing no other chemicals except liquid water at elevated temperature and pressure [6, 7]. LHW leads to increased cellulose accessibility and minimal production of potentially inhibitory products [8]. In LHW pretreatment, water acts as both a solvent and a catalyst accompanied with released organic acids from biomass to help disrupting the cell wall matrix [9]. The reduced biomass recalcitrance and enhanced enzymatic hydrolysis are achieved through several physicochemical changes to the biomass during pretreatment. Depending on the pretreatment severities, the major changes include the dissolution of hemicellulose, partial removal and relocation of lignin, limited deconstruction of cellulose, and minimal carbohydrate degradation. Hemicellulose is reported to be almost completely solubilized and deconstructed from biomass in hot water pretreatment at ~ 200 °C for 50 min [10]. Grénman et al. measured hemicellulose sugars extracted from softwood at 150–170 °C during LHW and reported that the dissolution of hemicellulose was highly dependent on the pretreatment temperature, while its degradation was strongly influenced by the pH of the liquid system [11]. In contrast to hemicellulose, cellulose has been less impacted by LHW pretreatment. Less than 22 *wt*% cellulose were degraded in wood and herbaceous biomass pretreated with LHW at 200 to 230 °C [12]. Kumar et al. analyzed biomass crystallinity using X-ray diffraction and indicated that controlled pH pretreatment significantly increased the biomass crystallinity of poplar [13]. Studies also revealed that lignin could migrate, coalesce, and solubilize at least partially at LHW pretreatment conditions and may redeposit from the solution onto biomass as the pretreated slurry cools down [14, 15]. These changes of biopolymers occurring during LHW pretreatment of biomass contributed more or less to the reduced biomass recalcitrance. More recently, the glass transition temperature of isolated lignin after LHW pretreatment was found to increase from 171 to 180 °C paralleling pretreatment severities and lignins from the more severely pretreated hardwood exhibited more pronounced enzymatic hydrolysis inhibition [16]. Although these physicochemical changes of biomass resulted from hydrothermal pretreatment provide insights into biomass recalcitrance [17], details in structural changes of cellulose, hemicellulose, and lignin in the molecular level with various LHW pretreatment severity have not been well understood.

Fast-grown poplar is a well-suited feedstock for a variety of applications such as bioenergy, pulp and paper, and bio-based materials [18]. After LHW pretreatment at 180 °C for 18–70 min, the pretreated poplar residues had significantly increased saccharification efficiency of 39–70% based on glucan and 35–57% based on xylan in comparison to the untreated poplar of 20% and 21%, respectively (Additional file 1: Table S1). To better understand the mechanism involved in hydrothermal pretreatment, we investigated the structural changes of hemicellulose, cellulose, and lignin of poplar in LHW pretreatment with different pretreatment severity factors ranging from 3.6 to 4.2 in this study.

## Results

### Chemical composition of untreated and LHW pretreated poplar

**Fig. 1:**
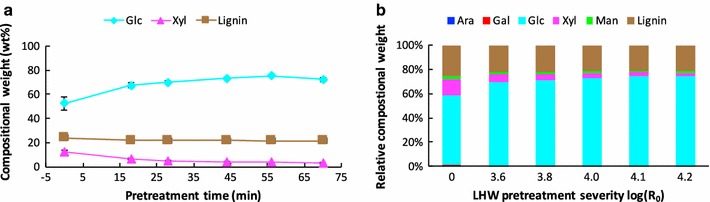
Chemical composition of untreated and LHW pretreated poplar. The *x*-axis denotes the pretreatment severity corresponding to different pretreatment time. *Ara* arabinose; *gal* galactose; *glc* glucose; *xyl* xylose; *man* mannose

The single-stage LHW pretreatment of poplar was conducted at 180 °C for five different cooking time of 18, 28, 44, 56, and 70 min which gave rise to severity factors (log *R*_0_) of 3.6, 3.8, 4.0, 4.1, and 4.2, respectively. The untreated poplar was designated a severity factor of 0. The compositions of LHW pretreated and untreated poplar are presented on the basis of dried solids (Fig. 1, the values of chemical composition are listed in Additional file 1: Table S2). Without pretreatment, the poplar is composed of, on a basis of dry matter, 23.8% lignin, 52.5% glucan, 12.3% xylan, 1.9% mannan, and small amounts of arabinan (0.4%) and galactan (0.7%). LHW pretreatment resulted in a significant dissolution of hemicellulose. For instance, the major component in poplar hemicellulose, xylan, decreased from 12.3 to 6.2% (*w/w*, dry matter) for 18 min and to 2.8% (*w/w*, dry matter) for 70 min; arabinan were completely solubilized and only a small amount of galactan was retained after 18 min pretreatment. Although substantially solubilized, 62% (*w/w*, dry matter) mannan remained even after 70 min pretreatment, which is consistent with the change in diluted acid pretreated poplar [19]. On contrary, cellulose (glucan) and lignin were mostly preserved in the solid residues after LHW pretreatment. Associated with the hemicellulose dissolution, the relative content of cellulose increased 28–38% (*w/w*, dry matter) upon the pretreatment severity from 3.6 to 4.2. However, the relative lignin content was slightly reduced from 23.7% (*w/w*, dry matter) in the untreated poplar to about 21.3% (*w/w*, dry matter) in the LHW pretreated poplar solids. This indicates that LHW pretreatment is not effective in lignin removal from biomass which is consistent with the literature results regarding hardwood pretreatment at 180–190 °C [16]. This non-effective lignin content removal was also reported in dilute acid pretreated poplar [19].

### Structural changes of cellulose in untreated and LHW pretreated poplar

**Fig. 2:**
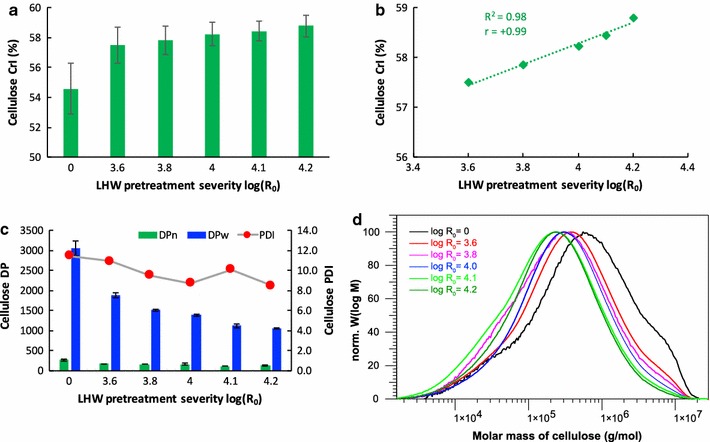
Cellulose structural changes with various LHW pretreatment severities. **a** cellulose CrIs measured by solid-state NMR; **b** linear regression and correlation of cellulose CrIs with pretreatment severities; **c** cellulose degree of polymerization (DP) and polydispersity index (PDI); **d** chromatograms of cellulose molecular weight measured by GPC. r is correlation coefficient value

The cellulose crystallinity index (CrI) and the degree of polymerization (DP) of cellulose have been measured to assess the effects of LHW pretreatment on poplar cellulose. The CrIs measured using solid-state NMR were in the range of 54.5–58.8% (Fig. 2). The cellulose crystallinity results are consistent with the reported results of *Populus* (54–63%) [20, 21]. In comparison to the untreated poplar, LHW pretreated poplar had slightly increased cellulose CrI along with the pretreatment severity likely due to the preferential deconstruction of the amorphous region of cellulose. The cellulose CrIs were positively dependent on and linearly correlated (*R* ^2^ = 0.98, *r* = + 0.99) with the investigated pretreatment severities (Fig. 2b).

The number-average degree of polymerization (DP_*n*_) and the weight-average degree of polymerization (DP_*w*_) of cellulose from untreated and LHW pretreated poplar were in the range of 100–300 and 1000–3000, respectively (Fig. 2c). The DP_*n*_ (266) and DP_*w*_ (3042) of the untreated poplar are comparable with the previous study [19] and the cellulose DPs of other poplar species reported by Meng et al. [18]. The GPC distribution curves of cellulose (Fig. 2d) revealed that LHW pretreatment had a significant impact on reducing cellulose molecular weight (chromatograms of pretreated samples in colors were shifted to the low-molecular-weight side compared with the untreated poplar in black). In comparison to the untreated poplar, the LHW pretreated solids had 35–53% and 38–65% reduction in cellulose DP_*n*_ and DP_*w*_, respectively. The polydispersity index (PDI) of cellulose was also reduced from 11.4 to 8.5 after the LHW pretreatment of poplar at log *R*_0_ of 4.2.

### Structural changes of hemicellulose in poplar after LHW pretreatment

**Fig. 3:**
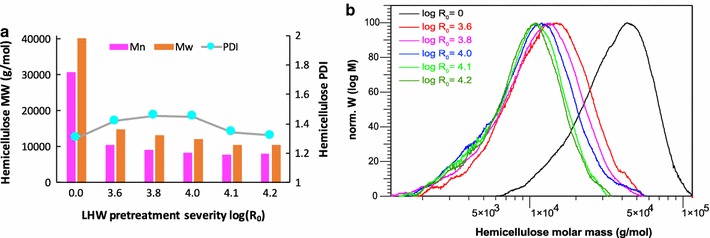
Average molecular weights of hemicellulose and the chromatographic distribution of hemicellulose molecular weight

The extracted hemicellulose from the untreated poplar had a number-average molecular weight (*M*_*n*_) of 3.1 × 10^4^ g/mol, a weight-average molecular weight (*M*_*w*_) of 4.0 × 10^4^ g/mol, and PDI of 1.3 (Fig. 3a). The *M*_*w*_ is consistent with the alkaline extracted hemicellulose reported by Sun et al. [22] and the *M*_*n*_ and PDI are consistent with hemicellulose extracted from poplar with ultrasound assistance [23]. Accompanying with the hemicellulose solubilization, the molecular weights of hemicellulose were considerably decreased (60–75%) after LHW pretreatment. The reduction of hemicellulose molecular size was dependent on the pretreatment severities. GPC profiles revealed that the hemicellulose extracted from LHW pretreated solid had a significantly shifted chromatographic distribution from the peak at ~ 4.5 × 10^4^ g/mol for the untreated control toward the smaller size centered at ~ 1.0 × 10^4^ g/mol (Fig. 3b).

## 2D HSQC (^13^C-^1^H) spectra elucidating lignin structural units and inter-unit linkages

**Fig. 4:**
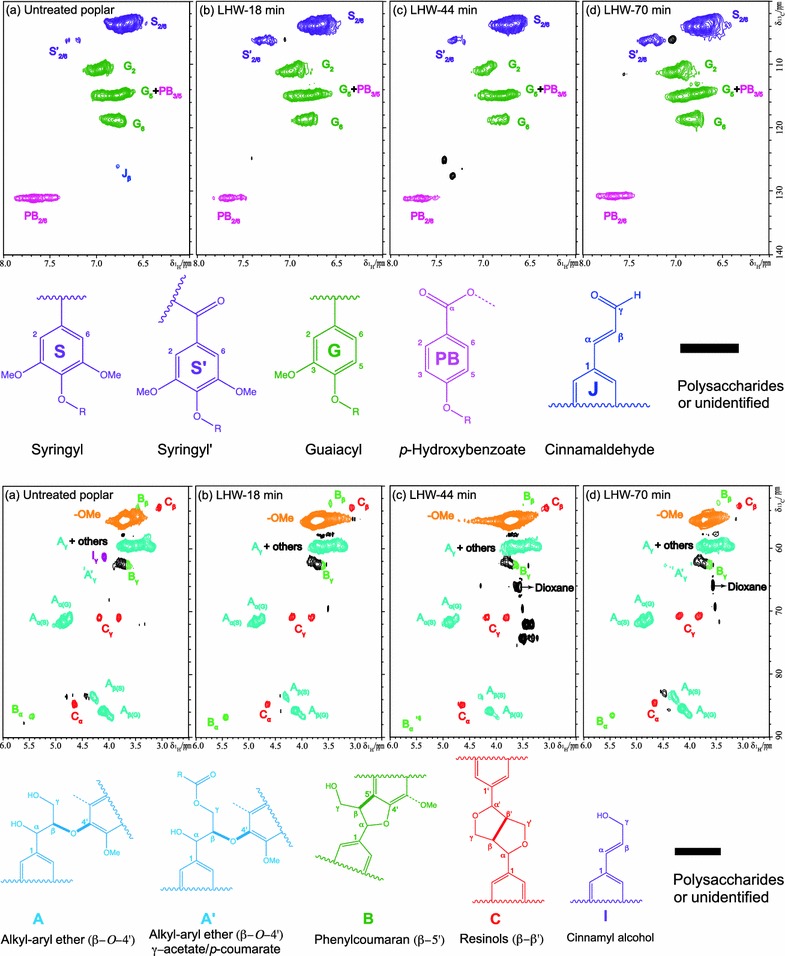
Short-range 2D NMR (HSQC) spectra revealing lignin subunits (top) and inter-unit linkages (bottom). Top: aromatic regions at *δ*_C_/*δ*_H_ 100-140/6.0-8.0 ppm; bottom: side chain regions at *δ*_C_/*δ*_H_ 50–90/2.5–6.0 ppm. Lignin subunits and inter-unit linkages are labeled with letters corresponding to given color-coded structures

The 2D HSQC NMR spectra of the lignin were compared in Fig. 4 with aromatic regions revealing lignin subunits and aliphatic regions revealing inter-unit linkages (cross-peak assignments were summarized in Additional file 1: Table S3). The LHW pretreatment of poplar for 18, 44, and 70 min corresponding to the severities of 3.6, 4.0, and 4.2, respectively, were presented to elucidate the lignin structural changes. The cross signals for various monolignols such as syringyl (S), guaiacyl (G), and *p*-hydroxybenzoate (PB) were well observed in the aromatic regions of HSQC spectra (Fig. 4 top). HSQC semi-quantitative estimation (Additional file 1: Table S4) showed that the LHW pretreated poplar had similar amounts of S/G ratio (1.1–1.2). After pretreatment, the intensity of cross peaks at *δ*_C_/*δ*_H_ 106.4/7.20 and 106.4/7.34 ppm assigned to oxidized syringyl units (S′) has been increased substantially. HSQC spectra also revealed that a small amount of cinnamaldehyde (J) contained in the untreated poplar lignin was not observed in the lignins from LHW pretreated poplar. In addition, LHW pretreatment resulted in a significant reduced amount of PB units in lignin, e.g., 7.9% for LHW-70 compared with 14.7% for the untreated poplar (Additional file 1: Table S4). However, the variation of monolignol levels (60.5, 61.1, and 63.4% S units and 39.5, 38.9, and 36.4% G units for LHW-18, 44, and 70 min, respectively) among the three LHW pretreated poplar lignin associated with different severities were comparable.

In the lignin side chain regions of HSQC spectra (Fig. 4 bottom), the cross peaks for methoxyl group (OMe) and alkyl–aryl ether β-*O*-4′ linkages dominated other signals (see detailed cross peaks assignments in Additional file 1: Table S3). Other inter-unit linkages, such as β-5′ in phenylcoumaran (B) and β-β′ linkages in resinol (C) substructures, were clearly detected in all the lignins. LHW pretreatment also had influence on the lignin side chain linkages shown in the HSQC aliphatic regions. For instance, the cinnamyl alcohol end group (I) contained in the untreated poplar has completely disappeared in the lignin after LHW pretreatment. In addition, semi-quantitative estimation of the cross signals revealed that the alkyl–aryl ether β-*O*-4′ linkages decreased 22% after LHW pretreatment (Additional file 1: Table S4). Similar to the substructures revealed in the aromatic regions above, the inter-unit linkages in lignin (5.7, 3.6, and 4.0% β-5′ and 3.0, 3.9, and 3.2% β-β′ for LHW-18, 44, and 70 min, respectively) did not have substantial changes upon the LHW pretreatment severities.

## ^13^C quantitative analysis of lignin from LHW pretreated poplar

**Fig. 5:**
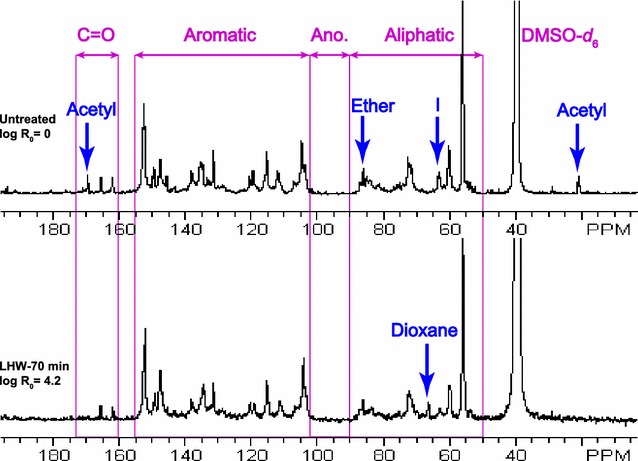
Quantitative ^13^C NMR spectra of cellulolytic enzyme lignins isolated from untreated (top) and LHW pretreated (bottom) poplar. *Ano* anomeric region of incorporated carbohydrates; *I* cinnamyl alcohol end group

**Fig. 6:**
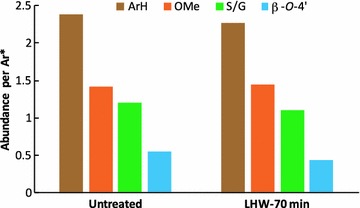
^13^C NMR quantitative analysis of lignin from untreated and LHW pretreated poplar. *Data presented were on a basis of per aromatic level except S/G ratio. *ArH* aromatic carbon; *OMe* methoxyl

Due to the similarity of HSQC spectra among the lignins (LHW-18, 44, and 70 min) from LHW pretreated poplar with different severities, LHW-70 min, the highest severity of our investigated conditions, was used to quantitatively assess the lignin structural changes after pretreatment (Fig. 5) as compared to the untreated poplar. The signal assignments and quantitative analysis of ^13^C NMR spectra of lignin were performed according to the published literatures [24–26]. The chemical shifts and peak assignments were listed in Additional file 1: Table S5. The ^13^C spectra of poplar lignin have been divided into four major regions—carbonyl (C=O) at 173–160 ppm, aromatic at 155–102 ppm, anomeric at 102–90 ppm, and aliphatic regions at 90–50 ppm from down-field to up-field (Fig. 5). The peaks in the carbonyl regions may be originated from aliphatic carboxylic and aliphatic esters. The aromatic regions denoted the aromatic carbons of lignin. Signals in the anomeric region revealed the anomeric carbon of incorporated or remained carbohydrates in the isolated lignin. The low level of peaks detected in the anomeric regions suggested that the isolated lignin contained very little carbohydrates. The aliphatic region denoting the inter-unit linkages showed the major changes of lignin structure in LHW pretreated poplar, such as decreased alkyl–aryl ether (β-*O*-4′) at 87–84 ppm, approximately diminished cinnamyl alcohol end group (I) at 61.6 ppm, which are consistent with the results observed from HSQC spectra above. In addition, the acetyl groups with methyl C at 20.7 ppm and carbonyl C at 169.4 ppm were almost completely removed in the LHW-70 min pretreated poplar. ^13^C NMR quantitative analysis revealed that LHW pretreatment resulted in 22% reduction of β-*O*-4′ ether linkage, whereas the levels of aromatic carbon, S/G ratio, and methoxyl groups were similar to the untreated poplar (Fig. 6).

## Discussion

### LHW resulted in a substantial dissolution of hemicellulose

**Fig. 7:**
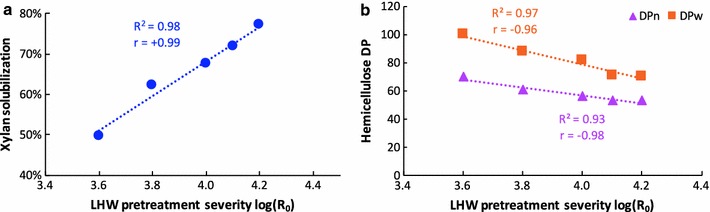
Linear regression and correlation of LHW pretreatment severity with xylan solubilization (left) and hemicellulose DP (right). r is correlation coefficient value

The chemical composition of biomass is an important factor for biomass utilization as well as its digestibility. A previous study has shown that the cellulose accessibility was strongly and negatively correlated with the presence of xylan content after pretreatment [27]. The authors reported ~ 200% increased orange dye adsorption (i.e., more cellulose accessibility to enzymes) for poplar pretreated at 160 °C for 10 min with LHW and the hot water pretreated poplar exhibited substantially increased sugar release and thus reduced biomass recalcitrance. In our study, the major hemicellulose component, xylan, has been solubilized more than 50% (*w/w*, dry matter) even at the least severe condition (180 °C, 18 min). As reported for LHW pretreated herbaceous and woody biomass, hemicellulose dissolution is one of the major factors contributing to the enhanced biomass porosity and enzymatic digestibility [9]. Recently, it has been reported that the presence and removal of hemicellulose and the LCC complex determines the nano-porous structures distribution in cell wall, which provides experimental data supporting the contribution of hindered accessibility to biomass recalcitrance [28]. Depending on the pretreatment severity factor, the behavior of hemicellulose is divided into three stages: the initial reaction on biomass surface, dissolution of fragmented hemicellulose, and further decomposition of carbohydrates in the solution [29]. One of the attractive sides from LHW was the high recovery of hemicellulose-derived sugars which could be utilized to add extra-values to the cellulosic ethanol production [30]. Although the amounts of inhibitors and their distribution depend on the type and severity of pretreatment, concentration of lignocellulosic solids during pretreatment, and biomass type, the solubilized hemicellulose mainly comprised of oligosaccharides with minimal degradation compounds (e.g., furfural and HMF) could be achieved at mild pretreatment severity with the absence of added mineral acids or alkaline catalyst [6, 31]. Our empirical results showed that the solubilization of xylan from poplar is strongly correlated (*r* = + 0.99) with the LHW pretreatment severity factors (Fig. 7a). Therefore, pretreatment severity factor could act as an important index to achieve a balance between high hemicellulose dissolution (increased cellulose accessibility) and raised sugars degradation (more inhibitory products).

In addition to the increased accessibility resulted from hemicellulose solubilization, the structure of the remaining hemicelluloses exhibited significantly reduced molecular weights (66–74%) after LHW pretreatment (Fig. 3). With increased pretreatment severity from 3.6 to 4.2, the DP of hemicellulose decreased linearly with strongly negative correlation (Fig. 7b). Hemicellulose with reduced DP tends to have shorter chains and more end-sites for exo-xylanases which is beneficial to the enzymatic digestibility of the pretreated biomass. When mixed enzymes of cellulases and xylanases are subject to the biomass, a faster hydrolysis of remaining xylan could further increase the cellulose accessibility for cellulases.

### Effects of pretreatment severity on cellulose structure

Hydrothermal pretreatment is one of the promising technologies to reduce biomass recalcitrance by breaking down plant cell wall matrix. Biomass recalcitrance has also been found to be associated with the structures of cellulose [32]. The crystallinity and DP of cellulose are shown to be important factors affecting the enzymatic hydrolysis efficiency of cellulosic substrate or lignocellulosic biomass [33]. The crystallinity increment after various pretreatment has also been measured by X-ray diffraction which suggests a preferential removal of amorphous portion of biomass during pretreatment process [34]. The crystallinity measured for the entire biomass using X-ray diffraction varied with the feedstock species used [13]. For instance, the crystallinity index of poplar increased from 50 to 54%, whereas the crystallinity of corn stover decreased from 50 to 45% after LHW pretreatment. The partially remained hemicellulose and a large portion of retained lignin in the pretreated biomass could interfere with the non-crystalline regions’ determination using X-ray diffraction by contributing to the amorphous fraction of the entire biomass. Thus, the crystal structure measured by this method would be difficult to interpret and compare if the accurate portions of hemicellulose and lignin are unknown in the pretreated biomass. To minimize the interference from hemicellulose and lignin, we measured the cellulose crystallinity of isolated cellulose after delignification and hemicellulose removal. The cellulose CrI measured by CP/MAS solid-state NMR revealed a 6–9% increase after LHW pretreatment and a positive correlation (r = + 0.99) of CrIs with pretreatment severities (Fig. 2). In line with the change on cellulose CrI, the DP_*n*_ and DP_*w*_ of the isolated cellulose decreased substantially (35–65%) after LHW pretreatment and the reduction of cellulose chain was dependent on the pretreatment severities. The previous study of the effect of diluted acid pretreatment on poplar showed an even more apparent reduction of cellulose DP (70–87%) [19]. The various depolymerization responses of cellulose to LHW and diluted acid pretreatment suggest that the hydrolytic scission of cellulose glycosidic bonds is highly associated with the pH of the solution. Compared with the diluted acid pretreated poplar at similar severity [19], LHW had a reduction of cellulose DP in a much milder manner. As a result, LHW can retain more polysaccharides for enzymatic hydrolysis and prevent the excessive degradation of carbohydrates. In addition, it appears that the cellulose in poplar has been preferentially hydrolyzed on the amorphous region during the LHW pretreatment, which retained the crystal regions as well as reduced length of cellulose chains. CrI of Avicel was found to be negatively related with hydrolysis rate by cellulases [35]. Recently, much more details have been revealed on the cellulase–cellulose interaction using molecular dynamics and free energy simulations [36]. In addition to support the reported mechanism that cellobiohydrolase selectively binds to hydrophobic surfaces of native cellulose [37], the authors demonstrated that there is a thermodynamic driving force for cellobiohydrolase to translate from hydrophilic surface of cellulose (usually more hydrophilic than hydrophobic surfaces in cellulose) to the preferred hydrophobic surface. In terms of the length of cellulose chain, the reduced DP of cellulose increased the number of reducing ends available for cellobiohydrolases to attack thereof enhanced saccharification [38]. Therefore, these results point out that LHW have a significant impact on cellulose molecular length which could be favorable to the enzymatic hydrolysis though comprised by the slightly increased cellulose crystallinity.

### Effects of pretreatment severity on lignin structure

Lignin, a heterogeneous polymer comprised of phenylpropene units linked primarily via alkyl–aryl ether and carbon–carbon bonds, is considered to be the most recalcitrant major component of plant cell walls [5]. Lignin restricts enzymatic digestibility of biomass mainly through (a) physical barrier, (b) inhibitory effect, and (c) LCC linkages. The transformation of lignin during hydrothermal pretreatment plays important roles in changing the biomass recalcitrance [17]. It has been observed that lignin mainly migrates and coalesces during LHW pretreatment and the chemical-structural changes are less severe compared with diluted acid pretreatment [15]. In consistent with the findings by other researchers [39, 40], the LHW pretreatment in our study predominantly leads to a decrease in β-*O*-4′ linkages and a removal of acetyl groups, whereas the S/G ratio remained relatively constant (Fig. 4, 5, and 6). In addition, the different pretreatment severity factors employed in this study had a little variation on lignin structures. These results suggest that LHW pretreatment had no significant changes on lignin substructures or preferential removal/condensation of S or G units, while favored removal of S unit revealed by decreasing S/G ratio was observed during dilute acid pretreatment [19]. The decreased β-*O*-4′ linkages indicated the fragmentation of lignin during the pretreatment which could facilitate the lignin migration.

## Conclusions

The chemical–structural alterations that occur as a result of LHW pretreatment are a substantial removal of hemicellulose, depolymerization of cellulose, slight modification of lignin within the poplar cell wall matrix, which together contributed to the increased biomass accessibility and reduced recalcitrance. The LHW pretreatment severity factors employed had an empirical linear correlation with the increased xylan solubilization (*r* = + 0.99), decreased xylan DP_*n*_ (*r* = − 0.96), and increased cellulose CrIs (*r* = + 0.99). The pretreatment severity factor could be an important index to balance the solubilization of hemicellulose and sugar degradation. The influence from LHW pretreatment on lignin molecular structure changes are revealed by 2D HSQC and ^13^C NMR. LHW resulted in a decrease in β-*O*-4′ linkages and PB units, removal of cinnamyl alcohol end group, and acetyl groups. The cleavage of β-*O*-4′ linkages indicated lignin fragmentation which could contribute to lignin migration and enhanced biomass porosity. These observed molecular changes of the major biopolymer components during LHW pretreatment provide fundamental information on addressing factors associated with cell wall recalcitrance during hydrothermal pretreatment.

## Methods

### Materials and chemicals

Poplar (*Populus trichocarpa*) used in this study was harvested at Oak Ridge National Laboratory, TN [19]. The biomass size was reduced in a Wiley mill to pass a 1 mm screen and then sieved to collect the fractions between 0.18 and 0.85 mm. The *p*-dioxane used in this study was distilled over sodium borohydride prior to use. Peracetic acid solution (32 *wt*% in dilute acetic acid), phenyl isocyanate (assay grade), and dichloromethane (HPLC grade) were purchased from Sigma-Aldrich (St. Louis, MO). Anhydrous pyridine (EMD, Millipore) was purchased from VWR. Cellulase C1794 from *Trichoderma sp*. (3–10 units/mg) and β-glucosidase from almonds (10–30 units/mg) were purchased from Sigma-Aldrich (St. Louis, MO). All the reagents and chemicals, unless otherwise noted, were used as received.

### Liquid hot water pretreatment (LHW)

All pretreatments were conducted as a single-stage pretreatment in a stirred tank reactor (1.0 L) glass lined Parr reactor (4520 Series) equipped with a 4842 temperature controller [19]. Extractives-free poplar chips (~ 5.5 g) was charged in the reactor with 100.0 mL preheated DI water (60 °C) with 5% solids loading (*w/w*, dry matter) and sealed. The reactor was heated in a fluidized sand bath set to 400 °C. The impeller speed was adjusted to 100 rpm and the mixture was heated at ~ 4 °C/min and held at 180 °C for designated residence time (18, 28, 44, 56, and 70 min). The ramping time from room temperature to 180 °C was 39 min. The combined pretreatment temperature (*T*) and time (*t*) investigated corresponded to different pretreatment severity (log R_0_) calculated by the equation below:

$$\log R_{0} = \log \left[ {t \times { \exp }\frac{T - 100}{\omega }} \right],$$

where the value of ω represents an activation energy associated with the pretreatment with the value of 14.75 [41]. After each pretreatment, the reactor was quenched in an icy bath and the cooled pretreated slurry was vacuum filtrated through Whatman No. 4 filter paper to recover the solid fraction, namely LHW pretreated poplar. The collected solid fractions were finally vacuum dried at 45 °C prior to further analysis. These pretreated poplars corresponded to varying pretreatment severity (log R_0_) from 3.6, 3.8, 4.0, 4.1, and 4.2, while unpretreated poplar was designated log *R*_0_ = 0.

### Chemical compositional analysis

The compositional analysis of the untreated and HWP poplar was performed in a a two-step hydrolysis according to the protocol developed by NREL (http://www.nrel.gov/docs/gen/fy08/42623.pdf). In detail, extractives were removed by adding ~ 5 g of biomass into an extraction thimble in a Soxhlet extraction apparatus. The extraction flask was filled with toluene/ethanol (2/1 by volume) and then refluxed at a boiling rate for 24 h. The extractives-free samples were air-dried and stored in a refrigerator. To measure the carbohydrate and lignin contents, extractives-free samples were treated with 72 *wt*% sulfuric acid at 30 °C for 1 h with glass rod stirring periodically and then diluted to 4 *wt*% using deionized water and subsequently autoclaved at 121 °C for another 1 h. The precipitate was filtered through a G8 glass fiber filter (Fisher Scientific, USA), dried, and weighed to obtain Klason lignin content. The resulting filtrate was diluted and injected into a high-performance anion exchange chromatograph with pulsed amperometric detection (HPAEC-PAD) using Dionex ICS-3000 (Dionex Corp., USA) with an electrochemical detector, a guard CarboPac PA1 column (2 × 50 mm, Dionex), a CarboPac PA1 column (2 × 250 mm, Dionex), a AS40 automated sampler, and a PC 10 pneumatic controller at room temperature. 0.002 m and 0.004 m NaOH was used as the eluent and postcolumn rinsing effluent, respectively. The total analysis time was 70 min, with a flow rate 0.4 mL/min. Calibration was performed with standard solutions of glucose, xylose, arabinose, mannose, and galactose, and fucose was used as an internal standard. These measurements were performed in duplicate and the results were reported as the average.

### Isolation of cellulose and hemicellulose

Cellulose and hemicellulose were isolated from untreated and LHW pretreated poplar according to the published procedures [33, 42]. The extractives-free samples were delignified by peracetic acid with 5.0 g loading per g biomass. The solution consistency was adjusted to 5% (*w/w*) with deionized (DI) water and the holopulping was conducted at room temperature for 24 h with magnetic stirring. The solid residue, designated as holocellulose, was washed with excessive DI water (Milli-Q water with resistivity 18.2 MΩ cm at 25 °C) and air-dried at room temperature for 24 h. A sub-portion of the air-dried holocellulose (100 mg) was consecutively extracted at 25 °C with 17.5% (*w/v*) NaOH solution (5.0 mL) for 2 h, followed by 8.75% (*w/v*) NaOH solution (10.0 mL) for an additional 2 h. The alkaline slurry was then filtered and rinsed with 5 mL of 1% (*w/v*) acetic acid leading to a liquid fraction and a solid residue. The solid residue, namely α-cellulose, was washed with an excess of DI water and air-dried for the analysis of cellulose DP after derivatization. The liquid fraction, rich in hemicellulose, was adjusted to pH 6–7 with anhydrous acetic acid. Hemicellulose was then precipitated by adding three volumes of 100% ethanol to the liquid fraction. Hemicellulose was then obtained by centrifugation at 8000 rpm (267π rad/s) for 5 min and freeze dried for 24 h.

### Lignin isolation

**Scheme 1:**
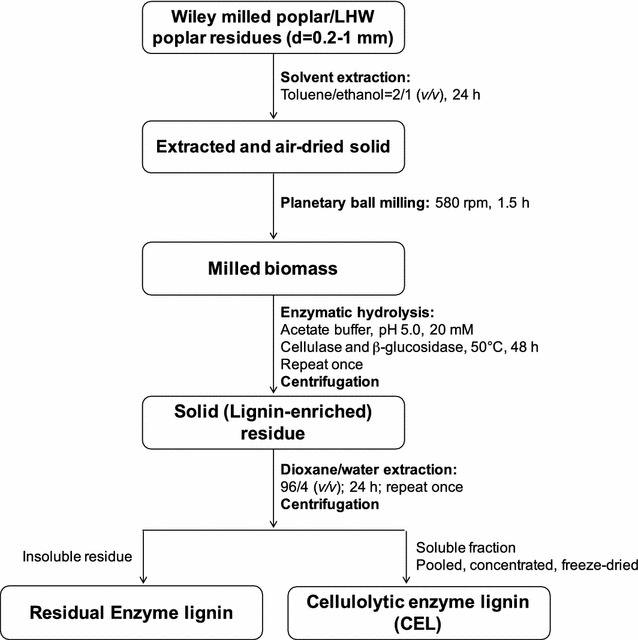
Cellulolytic enzyme lignin (CEL) isolation from untreated and LHW pretreated poplar

The cellulolytic enzyme lignin (CEL) was isolated from untreated and LHW pretreated poplar according to Scheme 1 [43, 44]. In brief, about 1 g the extractives-free sample was loaded to 50 mL ZrO_2_ grinding jar (including 10 × 10 ball bearings) in Retsch Ball Mill PM 100. The biomass was then ball milled at 580 RPM in a frequency of 5 min with 5 min pauses in-between for 1.5 h total time. The milled fine cell wall powder was then subjected to enzymatic hydrolysis with a mixture of cellulase and β-glucosidase (2;1, 5 *wt*% loading basis on cellulose weight) in acetic acid/sodium acetate buffer (pH 4.8, 50 °C) under continuous agitation at 200 rpm for 48 h. The residue was isolated by centrifugation and was hydrolyzed once more with freshly added enzymes. The residue obtained was washed with DI water (18.2 MΩ), centrifuged, and freeze dried, namely lignin-enriched residue. The lignin-enriched residue was extracted with dioxane-water (96% *v/v*, 10.0 mL/g biomass) for 24 h. The extracted mixture was centrifuged and the supernatant was collected. Dioxane extraction was repeated once by adding fresh dioxane-water. The extracts were combined, roto-evaporated to reduce the volume at less than 45 °C, and freeze dried. The obtained lignin samples, designated as CEL, were used for further analysis.

### Gel permeation chromatographic (GPC) analysis

The weight-average molecular weight (*M*_*w*_) and number-average molecular weight (*M*_*n*_) of cellulose were measured by GPC after tricarbanilation, as previously described [33, 42]. Briefly, the α-cellulose was derivatized with phenyl isocyanate in an anhydrous pyridine system prior to GPC analysis. Size-exclusion separation was performed on an Agilent 1200 HPLC system (Agilent Technologies, Inc, Santa Clara, CA) equipped with Waters Styragel columns (HR1, HR2, HR4, and HR6; Waters Corporation, Milford, MA). Number-average degree of polymerization (DP_*n*_) and weight-average degree of polymerization (DP_*w*_) of cellulose were obtained by dividing *M*_*n*_ and *M*_*w*_, respectively, by 519 g/mol, the molecular weight of the tricarbanilated cellulose repeating unit. The molecular weights of hemicellulose were measured by an Agilent 1200 series HPLC system equipped with three columns of Ultrahydrogel 120, 250, and 500 (Waters Inc.) linked in series according to [45]. The freeze dried hemicellulose samples were dissolved in 0.2 M sodium hydroxide/0.1 M sodium acetate (pH 11.8) mobile phase (~ 1.0 mg/mL) directly and filtered through a 0.45 µm filter before GPC analysis. Number-average degree of polymerization (DP_n_) and weight-average degree of polymerization (DP_*w*_) of hemicellulose were obtained by dividing *M*_*n*_ and *M*_*w*_, respectively, by 138 g/mol, the molecular weight of the xylose repeating unit.

$$M_{n} = \frac{{\mathop \sum \nolimits M_{i} *N_{i} }}{{\mathop \sum \nolimits N_{i} }}$$

$$M_{w} = \frac{{\mathop \sum \nolimits M_{i} *M_{i} *N_{i} }}{{\mathop \sum \nolimits M_{i} *N_{i} }}$$

$${\text{DP}}_{n} = \frac{{M_{n} }}{{M_{0} }}$$

$${\text{DP}}_{w} = \frac{{M_{w} }}{{M_{0} }},$$

where the *M*_*n*_ and *M*_*w*_ are number-average and weight-average molecular weight, respectively; DP_*n*_ and DP_*w*_ are number-average and weight-average degree of polymerization, respectively; N_i_ is the number of moles with the molar mass of *M*_*i*_; *M*_0_ is the molecular mass of repeating unit (519 g/mol in the case of derivatized cellulose; 132 g/mol in the case of hemicellulose).

### Solid-state NMR analysis

Solid-state NMR analysis for cellulose crystallinity was performed as previously described with minor modification [20, 33]. The isolated cellulose samples were stored in a sealed container to prevent moisture loss. The NMR samples were prepared by packing the moisturized cellulose into 4-mm cylindrical Zirconia MAS rotors. Cross-polarization magic angle spinning (CP/MAS) NMR analysis of cellulose was carried out on a Bruker Avance-400 MHz spectrometer operating at frequencies of 100.59 MHz for ^13^C in a Bruker double-resonance MAS probe head at spinning speeds of 8 kHz. CP/MAS experiments utilized a 5-µs (90°) proton pulse, 1.5-ms contact pulse, 4-s recycle delay, and 4000 scans. The cellulose crystallinity index (CrI) was determined from the areas of the crystalline and amorphous C_4_ signals using the following formula:

$${\text{CrI}} = \frac{{A_{{ 86-92 {\text{ppm}}}} }}{{A_{{ 86-92 {\text{ppm}}}} + A_{{ 79-86 {\text{ppm}}}} }}.$$

### NMR spectroscopic analysis

Nuclear magnetic resonance (NMR) spectra of isolated lignin samples were acquired in a Bruker Avance 400 MHz spectrometer and spectral processing used Bruker’s Topspin 3.5 (Mac) software. The ^13^C NMR acquisition was performed on a QNP probe using a 90° pulse with an inverse-gated decoupling pulse sequence, a 12-s pulse delay, and 12,288 scans at 50 °C. A standard Bruker heteronuclear single quantum coherence (HSQC) pulse sequence (hsqcetgp) was used on a BBFO probe with the following acquisition parameters: spectra width 10 ppm in F2 (^1^H) dimension with 2048 data points (acquisition time 256.1 ms), 210 ppm in F1 (^13^C) dimension with 256 increments (acquisition time 6.1 ms), a 1.5-s delay, a ^1^J_C–H_ of 145 Hz, and 32 scans. The central DMSO-*d*_6_ solvent peak (*δ*_C_/*δ*_H_ at 39.5/2.49) was used for chemical shift calibration. Relative abundance of lignin compositional subunits and inter-unit linkages were estimated semi-quantitatively using volume integration of contours in HSQC spectra [43, 46–48]. For monolignol compositions of S, G, H, and *p*-hydroxybenzoate (PB) quantitation, the S_2/6_, G_2_, H_2/6_, and PB_2/6_ were integrated. The Cα signals were used for contour integration for inter-unit linkages estimation.

## Additional file

**Additional file 1.** Supporting figures and tables.

## Abbreviations

CP/MAS: cross-polarization magic angle spinning
CrI: crystallinity index
DP_n_: number-average degree of polymerization
DP_w_: weight-average degree of polymerization
M_n_: number-average molecular weight
M_w_: weight-average molecular weight
