# Supplementary material for: The effect of liquid hot water pretreatment on the chemical–structural alteration and the reduced recalcitrance in poplar
Source: Biotechnol Biofuels. 2017 Nov 30;10:237. doi: 10.1186/s13068-017-0926-6 (PMC5707831; doi:10.1186/s13068-017-0926-6)
Supplement: Supplementary file 1 — Additional file 1. Supporting figures and tables. [file 13068_2017_926_MOESM1_ESM.docx]

Electronic Supporting Information

**The Effect of Liquid Hot Water Pretreatment on the Chemical Structural Alteration and the Reduced Recalcitrance in Poplar**

Mi Li ^1,2†^, Shilin Cao ^3†‡^, Xianzhi Meng^5^, Michael Studer ^1,4§^, Charles E. Wyman^1,4^, Arthur J. Ragauskas ^1,2,5,6*^, Yunqiao Pu ^1,2*^

*^1^ BioEnergy Science Center (BESC), USA*

*^2^ Biosciences Division, ORNL, Oak Ridge, TN, USA*

*^3^ School of Chemistry and Biochemistry, Georgia Institute of Technology, Atlanta, GA, USA*

*^4^ College of Engineering - Center for Environmental Research and Technology (CE-CERT), Bourns College of Engineering, University of California, Riverside, CA*

*^5^ Department of Chemical and Biomolecular Engineering, University of Tennessee, Knoxville, TN, USA*

*^6^ Department of Forestry, Wildlife, and Fisheries, Center for Renewable Carbon, University of Tennessee Institute of Agriculture, Knoxville, TN, USA*

This manuscript has been authored by UT-Battelle, LLC under contract no. DE-AC05-00OR22725 with the U.S. Department of Energy. The publisher, by accepting the article for publication, acknowledges that the United States Government retains a non-exclusive, paid-up, irrevocable, world-wide license to publish or reproduce the published form of this manuscript, or allow others to do so, for United States Government purposes. The Department of Energy will provide public access to these results of federally sponsored research in Accordance with the DOE Public Access Plan (<http://energy.gov/downloads/doe-public-access-plan>).

^†^ The two authors contributed equally to this work.

^‡^ S. Cao is currently at College of Material Engineering, Fujian Agriculture and Forestry University, Fuzhou, P. R. China.

^§^ M. Studer is currently at Laboratory for Bioenergy and Biochemicals, School of Agricultural, Forest and Food Sciences, Bern University of Applied Sciences, Switzerland.

***** Correspondence: Arthur J. Ragauskas, [aragausk@utk.edu](mailto:aragausk@utk.edu) ; Yunqiao Pu, [puy1@ornl.gov](mailto:puy1@ornl.gov).

**Table S1** Enzymatic hydrolysis results of glucan and xylan after LHW pretreatment

| Time (min) | Severity | Glucan% | Xylan% | Total% |
| --- | --- | --- | --- | --- |
| 0 | 0 | 20.2 | 9.8 | 20.6 |
| 18 | 3.6 | 38.7 | 19.8 | 35.1 |
| 28 | 3.8 | 38.5 | 19.9 | 34.7 |
| 44.0 | 4 | 64.2 | 17.4 | 52.8 |
| 56 | 4.1 | 64.3 | 14.7 | 52.3 |
| 70 | 4.2 | 70.1 | 15 | 57.4 |

Note: the yield presented was saccharification results based on the total glucan/xylan weight contained in the biomass/residues. Enzymatic hydrolysis conditions [1]: enzyme loadings were 15 + 5 to 105 + 35 mg of cellulase (Spezyme CP, protein content 116.0 mg/ml, Genencore, Palo Alto, CA) and xylanase (Multifect Xylanase, protein content 56.6 mg/ml, Genencore), respectively, per gram of glucan and xylan in the raw biomass. All of the resulting samples were incubated at 50°C in a shaking incubator at 150 with a throw of 25 mm (Multitron 2, Infors-HT, Bottmingen, Switzerland) for 72 hours.

**Table S2.** Original data used for Fig. 1: Chemical composition (wt%) of untreated and LHW pretreated poplar and its corresponded severity. The numbers in parenthesis are standard error of duplicates.

|  | Severity | Ara | Gal | Glc | Xyl | Man | Lignin |
| --- | --- | --- | --- | --- | --- | --- | --- |
| Untreated | 0 | 0.4(±0) | 0.7(±0.05) | 52.5(±5.39) | 12.3(±1.21) | 1.9(±0.85) | 23.8(±0.83) |
| 18 min | 3.6 | 0 | 0.2(±0.03) | 67.3(±2.21) | 6.2(±0.40) | 1.7(±0.38) | 21.6(±0.01) |
| 28 min | 3.8 | 0.0 | 0.1(±0.02) | 69.8(±1.24) | 4.7(±0.27) | 1.7(±0.18) | 21.8(±0.78) |
| 44 min | 4 | 0.0 | 0.1(±0.08) | 73.0(±0.43) | 4.0(±0.12) | 1.4(±0.27) | 21.6(±0.18) |
| 56 min | 4.1 | 0.0 | 0.1(±0.06) | 75.5(±0.96) | 3.5(±0.07) | 1.3(±0.15) | 21.4(±0.70) |
| 70 min | 4.2 | 0.0 | 0.1(±0.05) | 72.4(±1.69) | 2.8(±0.22) | 1.2(±0.11) | 21.3(±0.72) |

**Table S3** Assignments of the lignin ^13^C–^1^H correlation signals observed in the HSQC spectra of the lignins.

| Labels | δ_C_/δ_H_ (ppm) | Assignment |
| --- | --- | --- |
| B_β_ | 53.2/3.47 | C_β_/H_β_ in β-5' phenylcoumaran substructures (B) |
| C_β_ | 53.6/3.06 | C_β_/H_β_ in β-β' resinol substructures (C) |
| OMe | 55.5/3.71 | C/H in methoxyls |
| A_γ_ | 59.8/3.36 & 3.58 | C_γ_/H_γ_ in normal (γ-hydroxylated) β-O-4' substructures (A) |
| I_γ_ | 59.7/4.01? | C_γ_/H_γ_ in cinnamyl alcohol end-groups (I) |
| B_γ_ | 62.5/3.75 | C_γ_/H_γ_ in β-5' phenylcoumaran substructures (B) |
| A'_γ_ | 62.8/3.70 | C_γ_/H_γ_ in γ-acylated β-O-4' substructures (A′) |
| C_γ_ | 701.2/3.80 &4.17 | C_γ_/H_γ_ in β-β' resinol substructures (C) |
| A_α_/A'_α_ | 71.5/4.75& 71.9/4.89 | C_α_/H_α_ in β-O-4' substructures (A, A′) -G&-S, respectively |
| A'_β(G)_ | 83.0/4.23 | C_β_/H_β_ in γ-acylated β-O-4' substructures linked to a G unit (A′) |
| A'_β(S)_ | 83.3/4.33 | C_β_/H_β_ in γ-acylated β-O-4' substructures linked to an S unit (A′) |
| D_α_ | 81.4/4.74 | C_α_/H_α_ in 5-5' dibenzodioxocin substructures (D) |
| A_β(G)_ | 83.4/4.31 | C_β_/H_β_ in β-O-4' substructures linked to a G unit (A) |
| C_α_ | 85.2/4.63 | C_α_/H_α_ in β-β' resinol substructures (C) |
| A_β(S)_ | 85.8/4.09 | C_β_/H_β_ in β-O-4' substructures linked to an S unit (A) |
| B_α_ | 87.0/5.46 | C_α_/H_α_ in β-5' phenylcoumaran substructures (B) |
| S_2/6_ | 103.9/6.65 | C_2_/H_2_ and C_6_/H_6_ in etherified syringyl units (S) |
| S'_2/6_ | 106.4/7.26 | C_2_/H_2_ and C_6_/H_6_ in α-oxidized syringyl units (S′) |
| G_2_ | 110.9/6.95 | C_2_/H_2_ in guaiacyl units (G) |
| PB_3/5_ | 114.8/6.73 | C_3_/H_3_ and C_5_/H_5_ in *p*-hydroxybenzoate (PB) |
| G_5_ | 115.1/6.75 & 6.98 | C_5_/H_5_ in guaiacyl units (G) |
| G_6_ | 118.8/6.78 | C_6_/H_6_ in guaiacyl units (G) |
| PB_2/6_ | 131.4/7.68 | C_2_/H_2_ and C_6_/H_6_ in *p*-hydroxybenzoate (PB) |

**Table S4**. Relative abundance of lignin subunits and inter-units linkages (% presented on a basis of aromatic abundance S+G).

|  | Untreated | LHW-70 |
| --- | --- | --- |
| S% | 55.5 | 63.4 |
| G% | 44.5 | 36.6 |
| PB% | 14.7 | 7.9 |
| ArH% | 2.4 | 2.3 |
| OMe% | 1.4 | 1.4 |
| S/G | 1.24 | 1.13 |
| β-*O*-4'% | 55.4 | 43.6 |
| β-5'% | 3. 9 | 4.0 |
| β-β'% | 3.9 | 3.2 |

Note: S: syringyl, G: guaiacyl, PB: *p*-hydroxybenzoate; ArH: aromatic: OMe: methoxyl; S/G: ratio of syringyl over guaiacyl units.

**Table S5** Signal assignment in the ^13^C NMR quantitative analysis of lignin from BESC Poplar standard.

| δ_C_ (ppm) | Assignment |
| --- | --- |
| 169.4 | C=O in acetyl |
| 165.6 | C=O in PB |
| 162.0 | PB_4_ |
| 152.2 | S_3/5_ etherified |
| 149.3 | G_3_ etherified |
| 147.5 | G_4_ etherified |
| 147.0 | S_3/5_ non-etherified |
| 145.4 | G_4_ non-etherified |
| 137.9 | S_4_ etherified |
| 135.0 | S_1_/G_1_ etherified |
| 134.5 | S_1_/G_1_ etherified |
| 131.3 | PB_2/6_ |
| 128.6 | H_2/6_ |
| 121.1 | PB_1_ |
| 120.4 | H_1_ |
| 119.4 | G_6_ |
| 115.2 | G_5_ |
| 114.8 | PB_3/5_ |
| 111.8 | G_2_ |
| 104.6 | S_2/6_ |
| 87.0 | C_β_ in S β-*O*-4’ *threo* |
| 85.9 | C_β_ in S β-*O*-4’ *erythro* |
| 85.1 | C_β_ in G/H β-*O*-4’ *threo* |
| 83.9 | C_β_ in G/H β-*O*-4’ *erythro* |
| 72.4 | C_α_ in G/S β-*O*-4’ *erythro* |
| 71.3 | C_α_ in G/S β-*O*-4’ *threo* |
| 63.1 | C_γ_ in γ-acylated β-*O*-4’ |
| 61.6 | C_γ_ in I end group |
| 59.8 | C_γ_ in β-*O*-4’ |
| 56.0 | OMe in S/G |
| 53.7 | C_β_ in β-β’ |
| 52.2 | C_β_ in β-5’ |
| 20.7 | Acetyl |

**Table S6.** Data used for Fig. 2, Fig. 3, and Fig. 6. Cellulose crystallinity index (CrI), the number-average (DP_n_) and weight-average (DP_w_) degree of polymerization of cellulose and hemicellulose, the number-average (M_n_) and weight-average (M_w_) molecular weights of hemicellulose, and polydispersity index (PDI) of cellulose and hemicellulose. The numbers in parenthesis are standard error of duplicates.

| **Severity** | **Cellulose** | | | |  | **Hemicellulose** | | | | |
| --- | --- | --- | --- | --- | --- | --- | --- | --- | --- | --- |
|  | CrI% | DPn | DPw | PDI |  | Mn (g/mol) | Mw (g/mol) | DPn | DPw | PDI |
| **0** | 58.6  (±1.72) | 266  (±18) | 3042  (±181) | 11.44 |  | 30760 | 40138 | 205 | 267 | 1.30 |
| **3.6** | 57.5  (±1.20) | 172  (±0) | 1882  (±58) | 10.93 |  | 10510 | 14917 | 70 | 99 | 1.42 |
| **3.8** | 57.8  (±0.96) | 158  (±2) | 1505  (±28) | 9.52 |  | 9047.7 | 13159 | 60 | 88 | 1.45 |
| **4.0** | 58.2  (±0.77) | 159  (±24) | 1383  (±19) | 8.71 |  | 8409.7 | 12179 | 56 | 81 | 1.45 |
| **4.1** | 58.4  (±0.66) | 111  (±3) | 1116  (±46) | 10.07 |  | 7892.1 | 10610 | 53 | 71 | 1.34 |
| **4.2** | 58.8  (±0.71) | 125  (±15) | 1060  (±17) | 8.46 |  | 7980.9 | 10523 | 53 | 70 | 1.32 |

1. Studer MH, Brethauer S, DeMartini JD, McKenzie HL, Wyman CE. Co-hydrolysis of hydrothermal and dilute acid pretreated populus slurries to support development of a high-throughput pretreatment system. *Biotechnol Biofuels* 2011, 4(1):19.
